# Use of meat juice and blood serum with a miniaturised protein microarray assay to develop a multi-parameter IgG screening test with high sample throughput potential for slaughtering pigs

**DOI:** 10.1186/s12917-020-02308-4

**Published:** 2020-04-06

**Authors:** Katharina Loreck, Sylvia Mitrenga, Regina Heinze, Ralf Ehricht, Claudia Engemann, Caroline Lueken, Madeleine Ploetz, Matthias Greiner, Diana Meemken

**Affiliations:** 1 Institute for Food Quality and Food Safety, University of Veterinary Medicine Hannover, Foundation, Bischofsholer Damm 15, D-30173 Hannover, Germany; 2 Abbott (Alere Technologies GmbH), Löbstedter Straße 103-105, D-07749 Jena, Germany; 3 Department for Optical Molecular Diagnostics and Systems Technology, Leibniz-Institute of Photonic Technology (IPHT), Albert-Einstein-Straße 9, D-07745 Jena, Germany; 4 InfectoGnostics Research Campus, Centre for Applied Research, Philosophenweg 7, D-07743 Jena, Germany; 5 Institute of Physical Chemistry, Friedrich Schiller University Jena, Helmholtzweg 4, D-07737 Jena, Germany; 6 Indical Bioscience GmbH, Deutscher Platz 5b, D-04103 Leipzig, Germany; 7 LUFA Nord-West, Institut für Tiergesundheit, Ammerländer Heerstraße 123, D-26129 Oldenburg, Germany; 8 Department of Exposure, German Federal Institute for Risk Assessment (BfR), Max-Dohrn-Straße 8-10, D-10589 Berlin, Germany; 9 Institute of Food Safety and Food Hygiene, Section Meat Hygiene, Freie Universität Berlin, Königsweg 67, D-14163 Berlin, Germany

**Keywords:** Microarray, Serology, Pig, Abattoir, Zoonosis, Production disease, *Toxoplasma gondii*, *Yersinia enterocolitica*, Meat inspection, Food chain information

## Abstract

**Background:**

Serological screening of pig herds at the abattoir is considered a potential tool to improve meat inspection procedures and herd health management. Therefore, we previously reported the feasibility of a miniaturised protein microarray as a new serological IgG screening test for zoonotic agents and production diseases in pigs. The present study investigates whether the protein microarray-based assay is applicable for high sample throughput using either blood serum or meat juice.

**Material and methods:**

Microarrays with 12 different antigens were produced by Abbott (formerly Alere Technologies GmbH) Jena, Germany in a previously offered ‘ArrayTube’ platform and in an ‘ArrayStrip’ platform for large-scale use. A test protocol for the use of meat juice on both microarray platforms was developed. Agreement between serum and meat juice was analysed with 88 paired samples from three German abattoirs. Serum was diluted 1:50 and meat juice 1:2. ELISA results for all tested antigens from a preceding study were used as reference test to perform Receiver Operating Characteristic analysis for both test specimens on both microarray platforms.

**Results:**

High area under curve values (AUC > 0.7) were calculated for the analysis of *T. gondii* (0.87), *Y. enterocolitica* (0.97)*, Mycoplasma hyopneumoniae* (0.84) and *Actinobacillus pleuropneumoniae* (0.71) with serum as the test specimen and for *T. gondii* (0.99), *Y. enterocolitica* (0.94), PRRSV (0.88), *A. pleuropneumoniae* (0.78) and *Salmonella* spp. (0.72) with meat juice as the test specimen on the ArrayStrip platform. Cohens kappa values of 0.92 for *T. gondii* and 0.82 for *Y. enterocolitica* were obtained for the comparison between serum and meat juice. When applying the new method in two further laboratories, kappa values between 0.63 and 0.94 were achieved between the laboratories for these two pathogens.

**Conclusion:**

Further development of a miniaturised pig-specific IgG protein microarray assay showed that meat juice can be used on microarray platforms. Two out of twelve tested antigens (*T. gondii*, *Y. enterocolitica*) showed high test accuracy on the ArrayTube and the ArrayStrip platform with both sample materials.

## Background

The meat inspection of pigs in the European Union includes *ante-mortem* inspection, *post-mortem* inspection and food chain information (FCI) data. However, it is not possible to detect the most relevant pork-borne zoonotic hazards such as *Salmonella* spp., *Yersinia enterocolitica*, *Toxoplasma gondii* and *Trichinella* spp. [1] at the abattoir. The reason for this is that macroscopically visible lesions on the carcass or organs as well as clinical symptoms are largely absent in pigs infected with one or several of these pathogens. In the European Union, laboratory testing is compulsory for *Trichinella* ssp. (unless holdings are officially recognised as applying controlled housing conditions) and for *Salmonella* ssp. (according to Commission Regulation (EC) No 2073/2005). In addition, some EU countries have set up their own extended bacteriological or serological *Salmonella* ssp. monitoring programs. Meemken et al. [2] and Felin et al. [3] showed that continuous serological monitoring for more zoonotic agents could be a meaningful tool for risk categorisation of pig herds and enable targeted control measures at the abattoir. The advantages of serological testing include practicality, ease of sample collection and preparation, high sample throughput, low costs and fast turnaround times [4]. However, the serological examinations for multiple pathogens with available diagnostic methods for veterinary medicine (e.g. ELISA tests) would require an enormous amount of effort.

To overcome this obstacle, we previously reported the development of a miniaturised protein microarray as a new serological IgG screening test for zoonotic agents and production diseases in pigs [5]. Protein microarrays are excellently suited for the simultaneous detection of different analytes and have already been used for the simultaneous detection of different antibodies [6–8]. By coupling different antigens on the microarray chip surface, the respective antibodies can be detected in a joint test run, which not only saves costs, but also analysis time. The recently described microarray-based assay was produced with 12 different antigens and validated on ELISA pretested serum samples [5]. As respiratory pathogens such as the porcine reproductive and respiratory syndrome virus (PRRSV), *A. pleuropneumoniae* (APP) and *Mycoplasma (M.) hyopneumoniae* are of major economic concern in pork production worldwide [9], the antigen selection comprised not only zoonotic agents, but also antigens for these pig-specific pathogens. Antigens for two zoonotic agents (*T. gondii*, *Y. enterocolitica*) and three respiratory pathogens (APP, PRRSV, *M. hyopneumoniae*) showed highly promising test accuracy on the new microarray [5]. This result has encouraged the development of a pig-specific microarray including functionality for more antigens. However, the following two aspects are mandatory in order to enable the use of a microarray as a screening test in the field: First, the method should be suitable for high sample throughput, as serological monitoring for pathogens with unknown within-herd prevalence only makes sense if large sample sizes (e.g. annually 60 samples per herd for diseases with an within-herd prevalence of 5% [10]) can be examined. Second, the method should be applicable with meat juice as sample material. Meat juice sampling does not require interaction with live pigs, which is a clear advantage for animal welfare. Furthermore, meat juice sampling can be done cost-effectively by abattoir personnel. Sampling from the diaphragm pillar muscles is already known at the abattoirs for *Trichinella* ssp. sampling according to Commission Implementing Regulation (EU) No 2015/1375 and in Germany also from the national *Salmonella* ssp. monitoring program. This means existing logistics for meat juice sampling could be used and microarray analysis would provide added value to this sampling.

The microarray chip from the previous study was produced in the ‘ArrayTube’ platform offered by the manufacturer Abbot (Alere Technologies GmbH) Jena, Germany. A so-called ‘ArrayStrip’ platform was also produced by this manufacturer, which enables the analysis of microarrays in efficient 96-well plates. As meat juice samples have never been tested on the ArrayStrip platform, this study investigates whether the previously developed pig-specific microarray is compatible with meat juice samples and transferable to the ArrayStrip platform. Therefore, as first objective, test accuracy for the 12 different antigens was examined on both platforms with serum and meat juice as sample material. The second objective was to investigate whether applying serum or meat juice as sample material made a difference to the microarray results. As third objective, the applicability of the ArrayStrip platform was tested in three different laboratories for serum and meat juice to ensure comparability of results between different laboratories.

## Methods

### Reference samples

Meat samples (sized approximately 20 × 10 × 2 cm) from the diaphragm pillars were collected from 184 fattening pigs from 30 different pig herds between October 2016 and January 2017 at the slaughter line of three abattoirs located in an area with high pig density in the Northwest of Germany. The pigs were regularly delivered to the abattoirs at the end of the fattening period and sampling did not affect the release of carcasses for human consumption. The samples were frozen in plastic bags at minus 20 °C immediately after sampling and defrosted at 20 °C for collecting meat juice. Therefore, the plastic bags were hung up and a clamp was put underneath the meat, leaving just enough space on one side of the bag for the meat juice to drip to the bottom of the bag. This method was previously described by Meemken et al. [2] with an elastic plastic band instead of a clamp. After 12 h of defrosting, 6–12 mL meat juice from every bag could be transferred into plastic cups (Eppendorf AG, Hamburg, Germany) and stored at minus 80 °C until further analysis.

In parallel to the meat sampling, 184 blood samples from exactly the same pigs had been taken for the preceding study (ArrayTube platform with serum [5]). These blood serum samples were analysed with ten different ELISA tests (pigtype Toxoplasma Ab, pigtype Trichinella Ab, pigtype Yersinia Ab, pigtype Hepatitis E Virus Ab, pigtype Mycobacterium Ab, pigtype Swine Influenza Virus Ab, pigtype Salmonella Ab, pigtype PRRSV Ab (all Indical Bioscience GmbH, Leipzig, Germany), ID Screen *Mycoplasma hyopneumoniae* Indirect, ID Screen APP Screening Indirect serotypes 1–12 (both IDvet, Grabels, France)). A selection of 90 reference samples had been made for the preceding study, which optimally covered the measuring range of every ELISA test. The selection of reference samples was complemented with nine *Trichinella* spp. positive serum samples and individually matched meat juice samples from pig infection trials at the German National Reference Laboratory for *Trichinella* (German Federal Institute for Risk Assessment, Berlin, Germany), 20 *T. gondii* positive serum samples from MVZ Diamedis laboratory (MVZ Diamedis GmbH, Bielefeld, Germany) and seven *T. gondii* positive meat juice samples from infection trials at the Institute for Parasitology, University of Veterinary Medicine, Hannover, Germany. The additionally acquired seropositive samples for *Trichinella* and *T. gondii* were only used to characterise the respective *Trichinella* and *T. gondii* antigen on the microarray. The total number of negative and positive serum and meat juice samples for every antigen taken into account for the analysis of the different microarray platforms is displayed in the additional files (see Additional file 1).

### Microarray production

**Fig. 1:**
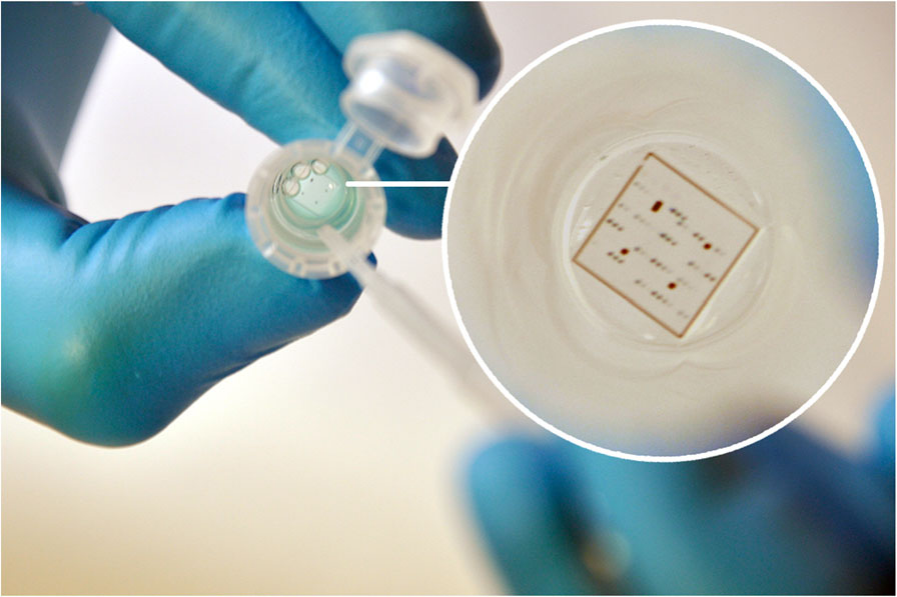
Processing of a microarray chip in the ArrayTube platform. The image in the white circle shows an enlarged image of the chip which is fixed to the bottom of the tube. The original size of the chip was 4.36 mm × 4.36 mm

**Fig. 2:**
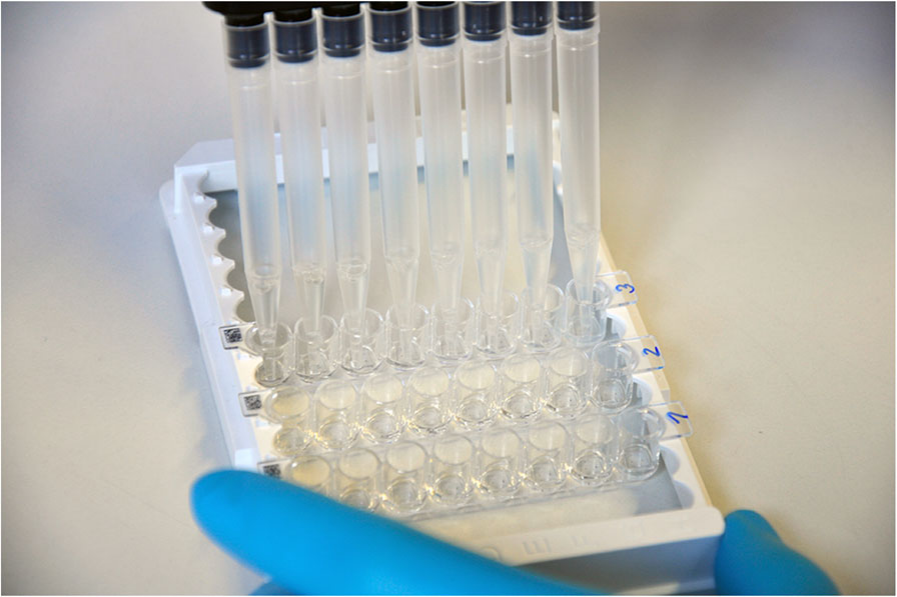
Processing of microarrays in the ArrayStrip platform. One strip consists of eight wells with a microarray chip (4.36 mm × 4.36 mm) attached to the bottom of every well. Strips can be processed individually or a maximum of 12 strips can be assembled on a 96-well frame. In this example, three strips were inserted into the frame

Two different microarray platforms (ArrayTube, ArrayStrip) manufactured by Abbott (formerly Alere Technologies GmbH) Jena, Germany were used in this study. In both platforms, the same microarray chip as produced for the preceding study [5] was attached to the bottom of the reaction vial (see Figs. 1 and 2).

The antigens were coupled to the glass surface of the microarray chip by means of an epoxy layer. The spotting and manufacturing process were previously described by Ehricht et al. [7]. The following 12 antigens from three different manufacturers were spotted and covalently immobilised as ‘antigen-spots’ on the chip: *T. gondii*, *Y. enterocolitica* mix, *Salmonella* spp. ELISA mix, *Salmonella* spp. in-house mix, *Trichinella* spp., *M. avium*, Hepatitis E virus, Influenza A virus, PRRSV in-house mix (all Indical Bioscience GmbH, Leipzig, Germany), *M. hyopneumoniae, A. pleuropneumoniae*, (both IDvet, Grabels, France) and *Y. enterocolitica* Yop O:3 (Institut Virion-Serion GmbH, Wuerzburg, Germany). The antigens offered by Indical Bioscience and IDvet were exactly the same antigens as used in producing the respective ELISA tests. More information on the antigens, the different antigen concentrations that were spotted and the layout of the chip is published in the preceding study [5]. Most antigen concentrations were spotted in quadruplicate and the median of replicated spots was established as test outcome for every antigen concentration. Purified-pig IgG (BIOMOL GmbH, Hamburg, Germany) was spotted to confirm a correct binding of the conjugate and the substrate on every microarray chip.

### Microarray test procedures

The test protocol for the analysis of serum on the ArrayTube platform is published in the preceding study [5]. In summary, antibodies that are present in the sample bind to the corresponding antigen spots during incubation time and all spots were antibodies are bound are detected by adding anti-pig-IgG-HRP conjugate, which is made visible by adding HRP-substrate.

In contrast to the test protocol with serum, the washing steps after sample incubation and after adding the conjugate had to be increased from three times to five times for applying meat juice on the microarray. The meat juice itself was centrifuged for 3 min at 4000 rpm immediately before preparing the sample dilution (1:2) from the meat juice supernatant. Different numbers of washing steps and different dilutions of meat juice were preliminarily tested.

For the analysis on the ArrayStrip platform, instead of 500 μL for pre-washing and 350 μL for all other washing steps, only 150 μL protein binding buffer were used in each washing step due to the smaller volume of the wells. Sample preparation for the ArrayStrip platform was identical to the ArrayTube platform: Blood samples had been centrifuged for 10 min at 2000 rpm on the day of sampling and the serum supernatant was diluted of 1:50. Meat juice was prepared as described before. Shaking of ArrayTubes and ArrayStrips was performed with horizontal thermoshakers (BioShake iQ, Quantifoil Instruments GmbH, Jena, Germany or PHMT Thermoshaker, Grant Instruments Ltd., Cambridge, United Kingdom). In order to aspirate liquids from a microarray, it is necessary to carefully approach the side of the tube with the tip of a pipette in order to avoid scratching the surface of the chip. Plastic transfer pipettes were used for this purpose on the ArrayTube platform and multi-channel pipettes on the ArrayStrip platform (see Figs. 1 and 2).

After aspirating the substrate from the microarrays, an image of every microarray was taken by the ArrayMate reading device (Abbott (Alere Technologies GmbH)). This device measures the intensity of staining from every spot on the microarray with a value between 0 (no signal, white spot) and 1 (maximum signal, black spot) as previously described [5]. Signal intensities between 0.1 and 0.7 are within the dynamic range of the test, a value below 0.1 cannot be assumed to show a correct antigen-antibody binding and a value above 0.7 indicates a color saturation of the spot [11]. Disruptive factors such as protein residues, scratches, dust or lint that are visible on the image, can result in an invalid measurement of one or several spots by the Iconoclust software (Abbot (Alere Technologies GmbH)) on the reading device.

### Microarray analysis in different laboratories

Three laboratories were involved in this study: The laboratory of the Institute for Food Quality and Food Safety, University of Veterinary Medicine Hannover, Germany, the laboratory of LUFA Nord-West, Oldenburg, Germany (accredited service laboratory affiliated with the Chamber of Agriculture in Lower Saxony, Germany) and the accredited food and veterinary service laboratory LVL Lebensmittel- und Veterinaerlabor GmbH, Emstek, Germany. In the following, the aforementioned laboratories are referred to as laboratories A, B and C. First, 106 meat juice samples were analysed on the ArrayTube platform in laboratory A. This included 90 meat juice samples originating from the same pigs as the serum samples that had been used for the development of the ArrayTube platform in the preceding study [5], 9 *Trichinella* seropositive and 7 *T. gondii sero*positive meat juice samples. The analysis of the ArrayStrip platform comprised 95 meat juice and 95 serum samples. This number was determined by the 96-well frame on which 95 samples could be analysed at one time, together with one sample that only contained the sample diluent buffer ‘pigtype blue’. This sample served as a control for false positive signals on antigen spots. The 95 samples consisted of 88 paired serum and meat juice samples from the analysis of the ArrayTube platform plus 7 *T. gondii* positive meat juice and 7 *T. gondii* positive serum samples that had also been used on the ArrayTube platform (no paired samples). As the seropositive *Trichinella* spp. samples did not show positive signals on the ArrayTube platform with serum and meat juice, the *Trichinella* antigen spots were not considered functional and no further *Trichinella* spp. seropositive samples were analysed on the ArrayStrip platform. Aliquots from the 95 serum and meat juice samples were sent to laboratories B and C together with all necessary processing liquids and ArrayStrips from the same printing lot as used in laboratory A. Both laboratories were equipped with ArrayMate reading devices and laboratory personnel had received training for microarray analysis from Abbott (Alere Technologies GmbH) together with laboratory A. Laboratories B and C analysed the meat juice and the serum samples on the ArrayStrip platform and submitted the microarray data to laboratory A for statistical analysis.

### Statistical analysis

Statistical analysis was performed with R version 3.6.1 [12] and Microsoft Excel 2010. To determine the accuracy of the antigens on the different platforms, receiver operating characteristic (ROC) curve analyses using the ELISA test results as reference were set up with the ‘pROC’ package [13] in R. Area under curve (AUC) confidence intervals were calculated by using the method by De Long [14]. Only antigens that had reached a minimum AUC value of 0.7 (moderate test accuracy [15]) for one of the spotted antigen concentrations were considered for further analysis. For these antigens, cut-off values were set as follows: First, the cut-off value was set to the maximum Youden Index [16]. If this resulted in a cut-off value below a signal intensity of 0.1, the minimum cut-off value 0.1 was chosen according to the dynamic range of the test. If setting the cut-off value to 0.1 implied a sensitivity or specificity below 0.6, the antigen concentration was excluded. Antigen concentrations that met these criteria were considered for agreement analysis between serum and meat juice as sample material. Therefore, Cohen’s kappa coefficients with 95% confident intervals were calculated with the ‘rel’ package [17] in R. In accordance with Landis and Koch [18] and Hunt [19], kappa values between 0.4 and 0.75 represent a fair to good agreement and those values higher than 0.75 an excellent agreement. In addition, Bland-Altman plots [20] were set up for a quantitative comparison between the measured signal intensities for paired serum and meat juice samples. Cohen’s kappa coefficients were also calculated for the level of agreement between the three laboratories.

## Results

### Test accuracy on ArrayTube and ArrayStrip platform

**Fig. 3:**
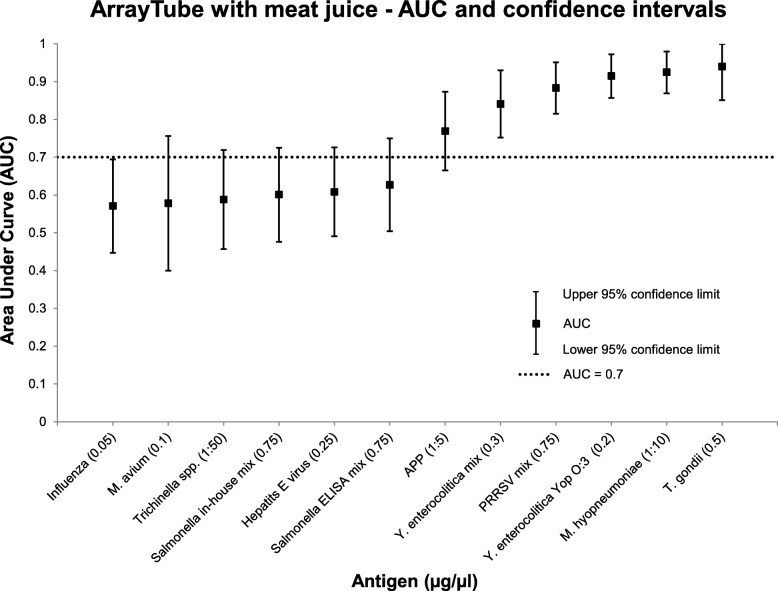
Area Under Curve (AUC) and 95% confidence limits for 12 different antigens examined with meat juice on the ArrayTube platform. Only the antigen concentration (μg/μL) which exceeded the highest AUC is shown. An AUC above 0.7 was reached for six different antigens

On the ArrayTube platform examined with meat juice, six different antigens reached an AUC above 0.7 (see Fig. 3). Regarding the cut-off values, the sensitivities and specificities (see Table 1), four of them (*T. gondii*, *Y. enterocolitica* Yop O:3, *M. hyopneumoniae*, APP) reached sensitivities between 75 and 92% and specificities between 61 and 98% with cut-off values set to 0.1 or higher. Table 1 shows the results of the five different antigen concentrations that showed the best results on both platforms with both sample materials. The full results of ROC analysis for all tested antigens and the different antigen concentrations are shown in the additional files (see Additional file 1). The measured microarray raw data are also shown in the additional files (see Additional file 2).

**Table 1:** Results of ROC analysis with cut-offs set in compliance with the technical abilities of the test

Antigen (concentration)	Microarray platform	Sample material	n (pos./neg.)^**1**^	AUC (95% CI)	Cut-off	Sensitivity	Specificity	Youden Index	Method cut-off
*T. gondii* (0.5)	ArrayTube	serum	110 (25/85)	0.91 (0.82–1)	0.11	0.84	0.95	0.79	Youden Max
	ArrayTube	meat juice	97 (12/85)	0.94 (0.85–1)	0.1	0.75	0.98	0.73	cut-off = 0.1
	ArrayStrip	serum	94 (12/82)	0.87 (0.73–1)	0.1	0.67	0.98	0.65	cut-off = 0.1
	ArrayStrip	meat juice	95 (12/83)	0.99 (0.98–1)	0.1	0.83	0.96	0.79	cut-off = 0.1
*Y. enterocolitica* Yop O:3 (0.5)	ArrayTube	serum	90 (39/51)	0.97 (0.93–1)	0.55	0.87	0.96	0.83	Youden Max
	ArrayTube	meat juice	90 (39/51)	0.91 (0.85–0.98)	0.34	0.92	0.84	0.76	Youden Max
	ArrayStrip	serum	87 (37/50)	0.97 (0.94–1)	0.29	1	0.94	0.94	Youden Max
	ArrayStrip	meat juice	88 (38/50)	0.94 (0.9–0.99)	0.24	1	0.78	0.78	Youden Max
*M. hyopneumoniae* (1:10)	ArrayTube	serum	90 (60/30)	0.93 (0.88–0.98)	0.42	0.83	1	0.83	Youden Max
	ArrayTube	meat juice	87 (58/29)	0.93 (0.87–0.98)	0.37	0.88	0.93	0.81	Youden Max
	ArrayStrip	serum	86 (56/30)	0.84 (0.75–0.92)	0.1	0.73	0.87	0.6	cut-off = 0.1
	ArrayStrip	meat juice	88 (58/30)	0.66 (0.55–0.78)	0.1	0.35	1	0.35	cut-off = 0.1
PRRSV in-house mix (0.75)	ArrayTube	serum	90 (64/26)	0.87 (0.8–0.94)	0.23	0.69	0.96	0.65	Youden Max
	ArrayTube	meat juice	90 (64/26)	0.88 (0.82–0.95)	0.1	0.44	1	0.44	cut-off = 0.1
	ArrayStrip	serum	87 (64/23)	0.69 (0.57–0.8)	0.19	0.42	0.96	0.38	Youden Max
	ArrayStrip	meat juice	88 (64/24)	0.88 (0.8–0.96)	0.1	0.55	0.96	0.51	cut-off = 0.1
APP (1:5)	ArrayTube	serum	90 (56/34)	0.75 (0.64–0.85)	0.17	0.61	0.59	0.2	Se Min 0.6
	ArrayTube	meat juice	89 (56/33)	0.77 (0.67–0.87)	0.17	0.8	0.61	0.41	Youden Max
	ArrayStrip	serum	87 (56/31)	0.71 (0.6–0.82)	0.1	0.54	0.87	0.41	cut-off = 0.1
	ArrayStrip	meat juice	87 (55/32)	0.78 (0.68–0.87)	0.1	0.6	0.84	0.44	Se Min 0.6

^1^ Number of positive and negative samples in ROC analysis

**Fig. 4:**
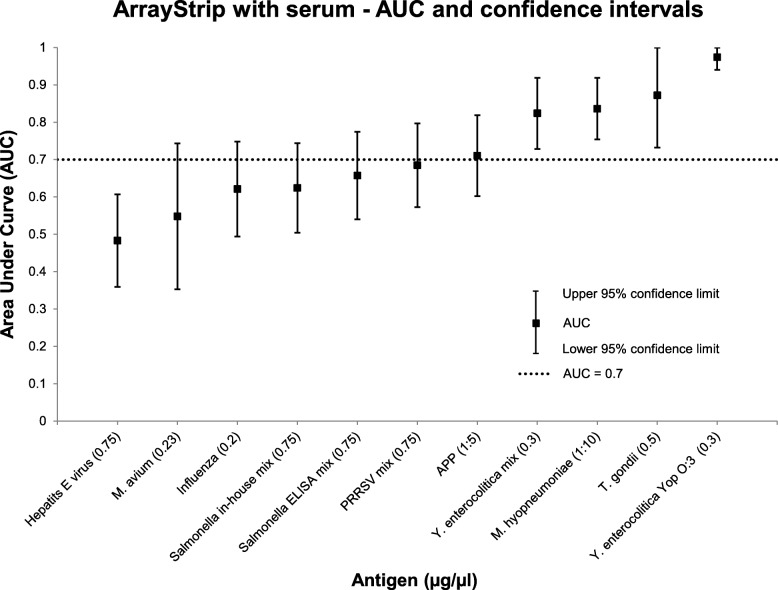
Area Under Curve (AUC) and 95% confidence limits for 12 different antigens examined with serum on the ArrayStrip platform. Only the antigen concentration (μg/μL) which exceeded the highest AUC is shown. An AUC above 0.7 was reached for five different antigens

On the ArrayStrip platform examined with serum, five different antigens reached an AUC above 0.7 (see Fig. 4). Three of them (*T. gondii*, *Y. enterocolitica* Yop O:3, *M. hyopneumoniae*) reached sensitivities between 67 and 100% with cut-off values set at 0.1 or higher (see Table 1). One microarray image from the 96-well frame was excluded from analysis because of no positive staining of the purified-pig IgG control spots.

**Fig. 5:**
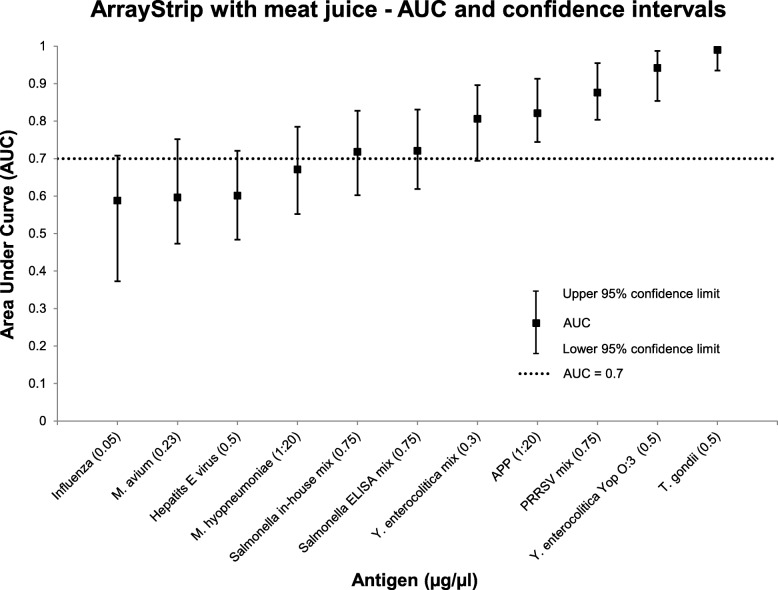
Area Under Curve (AUC) and 95% confidence limits for 12 different antigens examined with meat juice on the ArrayStrip platform. Only the antigen concentration (μg/μL) which exceeded the highest AUC is shown. An AUC above 0.7 was reached for seven different antigens

On the ArrayStrip platform examined with meat juice, seven different antigens reached an AUC above 0.7 (see Fig. 5). This also applied to the *Salmonella* ELISA mix and the *Salmonella* in-house mix antigen in the concentration 0.75 μg/μL. For these antigens, sensitivities of 60 and 73% and specificities of 78 and 69% were reached with a cut-off set at 0.11 for the ELISA mix and 0.16 for the in-house mix. The two antigens *T. gondii* and *Y. enterocolitica* Yop O:3 were considered for further agreement analysis between meat juice and serum.

### Agreement between serum and meat juice

**Fig. 6:**
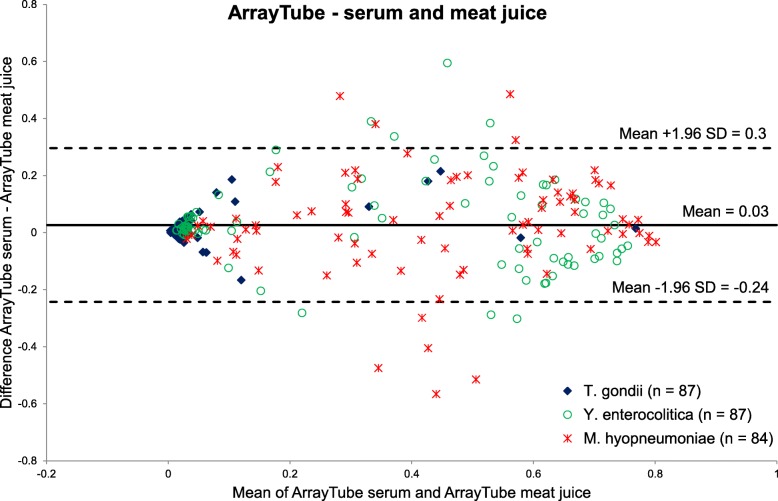
Bland-Altman plot for serum and meat juice analysis of three antigens (*T. gondii*, *Y. enterocolitica* Yop O:3, *M. hyopneumoniae*) on the ArrayTube platform

Table 2 shows the level of agreement between serum and meat juice on the ArrayTube platform for *T. gondii*, *Y. enterocolitica* Yop O:3 and *M. hyopneumoniae*. In order to calculate Cohen’s kappa values, results were dichotomised with the cut-off values presented in Table 1. High Cohen’s kappa values were reached for all three antigens on the ArrayTube platform. The Bland-Altman plot analysis for the measured signal intensities for serum and meat juice showed that only few values measured for *Y. enterocolitica* and *M. hyopneumoniae* exceeded the agreement levels (see Fig. 6).

**Table 2:** Contingency tables with Cohen’s kappa (κ) values (95% CI) for paired serum and meat juice samples analysed on the ArrayTube platform. Samples originate from 87 pigs slaughtered in 3 German abattoirs between October 2016 and January 2017

***T. gondii***				
κ = 0.69 (CI: 0.4–0.98)		ArrayTube meat juice		
		positive	negative	total
ArrayTube serum	positive	5	3	8
	negative	1	78	79
	total	6	81	*n* = 87
***Y. enterocolitica*Yop O:3**				
κ = 0.72 (CI: 0.58–0.87)		ArrayTube meat juice		
		positive	negative	total
ArrayTube serum	positive	32	2	34
	negative	10	43	53
	total	42	45	*n* = 87
***M. hyopneumoniae***				
κ = 0.66 (CI: 0.5–0.83)		ArrayTube meat juice		
		positive	negative	total
ArrayTube serum	positive	40	5	45
	negative	9	30	39
	total	49	35	*n* = 84

**Fig. 7:**
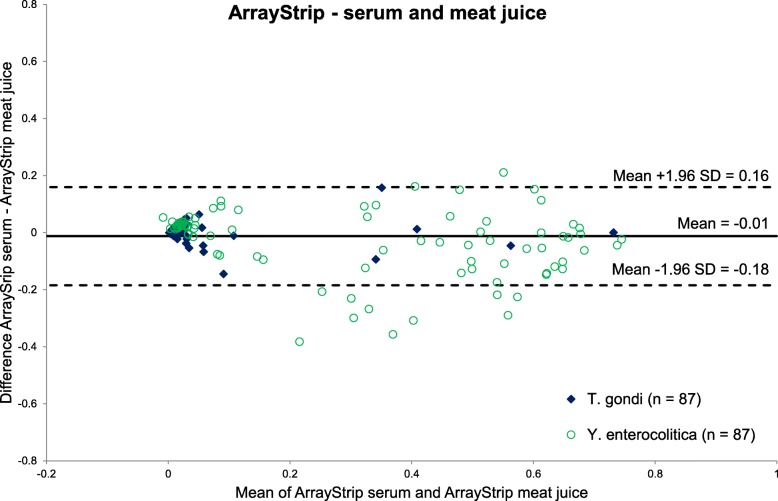
Bland-Altman plot for serum and meat juice analysis of two antigens (*T. gondii*, *Y. enterocolitica* Yop O:3) on the ArrayStrip platform

Table 3 shows the contingency tables and kappa values for *T. gondii* and *Y. enterocolitica* Yop O:3 examined on the ArrayStrip platform. Excellent agreement between serum and meat juice was reached for both antigens. In the corresponding Bland-Altman plot, the measured values stayed mainly within the levels of agreement (see Fig. 7). For *Y. enterocolitica*, a few more values could be found below the lower level of agreement. This shows that the measured values for meat juice tended to be slightly higher than for serum on the ArrayStrip platform, especially if higher signal intensities were measured.

**Table 3:** Contingency tables with Cohen’s kappa (κ) values (95% CI) for paired serum and meat juice samples analysed on the ArrayStrip platform. Samples originate from 87 pigs slaughtered in 3 German abattoirs between October 2016 and January 2017

***T. gondii***				
κ = 0.92 (CI: 0.75–1)		ArrayStrip meat juice		
		positive	negative	total
ArrayStrip serum	positive	6	0	6
	negative	1	80	81
	total	7	80	*n* = 87
***Y. enterocolitica*Yop O:3**				
κ = 0.82 (CI: 0.7–0.94)		ArrayStrip meat juice		
		positive	negative	total
ArrayStrip serum	positive	40	0	40
	negative	8	39	37
	total	48	39	*n* = 87

### Application of the ArrayStrip platform in different laboratories

The ArrayStrip platform was successfully tested in all three laboratories for both test specimens. Only one meat juice sample could not be processed by the reading device in laboratory B due to too many protein residues on the chip surface. On the single microarray chip that was examined with the sample diluent buffer on every 96-well frame serving as a control, no staining of spots was observed, except for the purified-pig IgG control spots. No microarrays examined in laboratories B and C had to be excluded due to a lack of staining on IgG spots. Regarding the mean of standard deviations among repeated spots, very low values were reached in all three laboratories (see Table 4). The percentage of invalid measurements was higher with meat juice than with blood serum as sample material in all three laboratories.

**Table 4:** Percentage of invalid measurements and mean of standard deviations among repeated spots measured on the ArrayStrip platform in the 3 different laboratories (A, B, C)

Laboratory	sample material	% invalid measurement	mean of SD^3^ among repeated spots
A	serum^1^	1	0.02
	meat juice^2^	4.9	0.02
B	serum^1^	0.2	0.01
	meat juice^2^	8.1	0.01
C	serum^1^	1	0.01
	meat juice^2^	6.1	0.02

^1^ Antigen spots of *T. gondii* 0.5, *Y. enterocolitica* 0.5 and *M.hyopneumoniae* 1:10 were included in the analysis

^2^ Antigen spots of *T. gondii* 0.5, *Y. enterocolitica* 0.5 and APP 1:5 were included in the analysis

^3^ The standard deviation (SD) among replicates was calculated from every microarray chip and the mean of these SD values is presented in the table

The qualitative comparison of results, based on the cut-offs presented in Table 1, showed contradicting Cohen’s kappa values related to the tested antigen (see Table 5). High agreement between the three laboratories was observed for the antigens *T. gondii* and *Y. enterocolitica* but no agreement could be shown for *M. hyopneumoniae* or APP.

**Table 5:** Cohen’s kappa (κ) values (95% CI) for serum and meat juice analysis on the ArrayStrip platform in 3 laboratories (A, B, C)

**ArrayStrip serum**		A	B	C
Lab.	Antigen	n ^1^	κ (95% CI)	κ (95% CI)
A	*T. gondii*		0.94 (0.83–1)	0.73 (0.47–0.98)
	*Y. enterocolitica*	x	0.68 (0.53–0.82)	0.79 (0.66–0-91)
	*M. hyopneumoniae*		0.02 (−0.02–0.06)	0.43 (0.25–0.6)
B	*T. gondii*	*n* = 94		0.64 (0.35–0.93)
	*Y. enterocolitica*	*n* = 94	x	0.63 (0.47–0.79)
	*M. hyopneumoniae*	*n* = 93		0.03 (− 0.03–0.1)
C	*T. gondii*	*n* = 94	*n* = 95	
	*Y. enterocolitica*	*n* = 94	*n* = 95	x
	*M. hyopneumoniae*	*n* = 93	*n* = 95	
**ArrayStrip meat juice**		A	B	C
Lab.	Antigen	n ^1^	κ (95% CI)	κ (95% CI)
A	*T. gondii*		0.81 (0.63–0-99)	0.85 (0.69–1)
	*Y. enterocolitica*	x	0.93 (0.86–1)	0.75 (0.62–0.88)
	APP		−0.02 (−0.06–0.02)	0.05 (− 0.07–0.16)
B	*T. gondii*	*n* = 94		0.84 (0.66–1)
	*Y. enterocolitica*	*n* = 94	x	0.75 (0.62–0.88)
	APP	*n* = 93		−0.03 (− 0.06–0)
C	*T. gondii*	*n* = 95	*n* = 94	
	*Y. enterocolitica*	*n* = 95	*n* = 94	x
	*APP*	*n* = 94	*n* = 94	

^1^*n* = Number of valid microarray results considered in the comparison between the laboratories

## Discussion

Official meat inspection procedures in the European Union are moving from a traditional, macroscopic examination of slaughtered animals towards a more risk based safety assurance system [21]. In view of this development, serological screening for zoonotic agents in pig herds is considered as a potential tool to improve food safety [2, 3, 22]. Felin et al. [3] stated that the best timing for serological screening is at the end of the fattening period or at the abattoir. This study investigated whether the specific demands of a screening test for pigs at the abattoir, including different sample materials and fast turn-around times, could possibly be met by using a protein microarray. Our results showed an assay that allows 96 pig meat juice or serum samples to be multi-serologically examined in less than 2.5 h. However, for using a multiple monitoring tool like a microarray in the field, the surveillance strategy has to comply with every pathogen individually, as it was recently described for the risk-based surveillance of different food-borne parasites by Felin et al. [23]. Regarding meat-borne zoonoses, it is important to consider whether interventions on farm level could possibly lower the prevalence or if risk-based action would be more effective at the abattoir for example concerning further processing of the meat.

Regarding test accuracy of the antigens on the ArrayTube and the ArrayStrip platform, nearly half of the spotted antigens reached medium to high AUC values. However, some antigens only showed low ability to discriminate although all antigens had been validated with reference sera by the antigen manufacturers for the production of ELISA tests previously. Therefore, it must be assumed that antigen standard formulations developed for the production of ELISA tests are not universally applicable on the microarray. Since it is not possible to predict to what extent 3D structures of the antigens are influenced by the coupling via the epoxy group on the microarray chip, it would be necessary to test more and different antigen formulations in order to achieve better results with these antigens on the microarray.

For *T. gondii* and *Y. enterocolitica*, the sensitivities and specificities reached on the microarray are close to values reported for commercially available ELISA tests. Steinparzer et al. [24] observed sensitivities ranging from 0.57 to 0.65 and specificities of 0.97 to 0.99 on three different *T. gondii* serum ELISA tests using microscopic agglutination as reference. Meemken et al. [2] stated a sensitivity and specificity of 1 for a commercially available *Y. enterocolitica* ELISA test used with serum or meat juice.

When evaluating sensitivities and specificities on the different microarray platforms, it is important to consider that results of ELISA tests performed with serum as sample material were taken as reference for ROC analysis for serum as well as for meat juice. In principle, ROC analysis requires the true status to be determined by a reference or gold standard test [25], as ELISA tests cannot be assumed to have perfect sensitivity and specificity. This may lead to bias (underestimation) in the evaluation of accuracy estimates of the microarray when using them as gold standard. However, in the absence of an available gold standard test, the comparison with the widely used ELISA tests has been found adequate. In our study, standard reference meat juice and serum samples containing antibodies of known concentration for all ten pathogens were not available and the paired samples offered the opportunity to compare microarray performance of serum and meat juice.

Test accuracy on the ArrayTube platform was only analysed for development purposes. For use as a herd test at the abattoir, only the ArrayStrip platform would be considered. Limits of 0.7 for the AUC and 0.6 for sensitivity and specificity as well as cut-offs in Table 1 were only preliminarily set for the agreement analysis of serum and meat juice in this study. For a full validation, in accordance with the principles of validation of diagnostic assays by the World Organisation for Animal Health (OIE) [26], cut-off values would have to be set depending on the purpose of the tested antigen and the epidemiological situation [27]. The fact that the antigen concentration which exceeded the highest AUC was mostly the highest antigen concentration that had been spotted on the chip and that the minimum of a cut-off value of 0.1 had to be selected several times (see Table 1), indicate that higher antigen concentrations need to be spotted and/or antigen formulations need to be modified in order to achieve higher signal intensities on positive samples. This was especially noticeable on the ArrayStrip platform.

The method used to sample meat juice in this study is not the usual way meat juice is sampled at the abattoir; for example, for the ELISA tests performed for national *Salmonella* monitoring in Germany or the muscle pieces that are sampled from the diaphragm pillars for direct *Trichinella* inspection based on microscopy. For ELISA analysis, standard meat juice funnels (Kabe Labortechnik GmbH, Nuembrecht, Germany) are used, but these funnels would not have contained sufficient volume for the repeated microarray tests performed in this study. The muscle pieces sampled in this study were larger and potentially contained more fat and sinewy parts than standard meat juice samples. This could have biased the meat juice in containing comparatively fewer antibodies and more protein and fat residues. Nevertheless, good agreement between meat juice and blood serum was observed on the ArrayTube platform (κ = 0.66 to κ = 0.72) and excellent agreement on the ArrayStrip platform (κ = 0.82 and κ = 0.92).

Previously, meat juice was discussed as not being a homogenous serological matrix [28]. In another study, where paired blood and diaphragmatic muscle samples were analysed with a commercial *Salmonella* ELISA, significantly higher optical density percentages were measured for serum [29]. Nevertheless, usability of meat juice for serological analysis of pigs has already been proven in many cases for different pathogens, for example for *Salmonella* spp. [30], PRRSV [31], Hepatitis E virus [32] and *T. gondii* [33].

Assuming that the antibody concentration in serum is around ten times higher than in meat juice [30, 31, 34], in our study, the microarray was first tested with a meat juice dilution of 1:10, due to an applied serum dilution of 1:100. This resulted in signal intensities that were far too low for the dynamic range of the microarray. Therefore, the sample dilution was increased to 1:2. This might be related to the increase in washing steps from three to five that had been necessary for the meat juice protocol. This decision was required as too much protein residue was visible on the microarray images when applying only three washing steps with meat juice, which resulted in images that could not be analysed by the reading device.

The Bland-Altman plots showed that the high level of agreement between serum and meat juice was also present in the quantitatively measured signal intensities. This confirms that the sample dilutions were chosen appropriately. For the ArrayTube platform, the mean value of differences was slightly positive (0.03) and for the ArrayStrip platform it was slightly negative (− 0.01). Therefore, no clear tendency for higher signal intensities can be derived for one of the two sample materials.

With regard to the application of the ArrayStrip platform in the three laboratories, the partially low kappa values indicate that reproducibility of the method might be dependent on the accuracy of the antigen. For serum as well as for meat juice the antigen which had reached comparatively low sensitivities and specificities (*M. hyopneumoniae* for serum and APP for meat juice) also showed the lowest kappa values. Further analysis of reproducibility is needed to trace the reasons for this.

The percentage of invalid measurements in the laboratories correspond to the percentages of missing values for microarray data that have been described to be usually higher than 5% [35] or to vary between 0.8 and 10% [36]. The higher percentage of invalid measurements for meat juice might be attributed to protein and fat residues in the sample material.

## Conclusions

A newly developed miniaturised pig-specific IgG protein microarray assay could successfully be applied with meat juice as sample material. The microarray is transferable to the ArrayStrip platform, which enables a multi-serological analysis of 96 samples in less than 2.5 h. From 12 different tested antigens, two antigens (*T. gondii* and *Y. enterocolitica*) showed high test accuracy on both tested platforms (ArrayTube and ArrayStrip) with both test specimens. Regarding these antigens, agreement between serum and meat juice was very high (kappa = 0.92 for *T. gondii*, kappa = 0.82 for *Y. enterocolitica*) when tested on the ArrayStrip platform with paired samples. Therefore serum and meat juice could be used interchangeably with this method. In a first comparative test of the ArrayStrip platform between three different laboratories, kappa values above 0.6 were straightforwardly reached for *T. gondii* and *Y.* enterocolitica. However, higher test accuracy for more antigens has to be achieved in order to increase the efficiency of the microarray in comparison to performing the existing single ELISA tests. Overall, it was shown that microarray technology offers ideal prerequisites as a diagnostic and surveillance tool to improve animal health as well as food safety.

## Supplementary information

**Additional file 1.** Results of ROC analysis for ArrayTube with meat juice and ArrayStrip with meat juice and serum for all tested antigens in all tested antigen concentrations. Cut-offs were set at the maximum Youden Index.

**Additional file 2.** Measured microarray raw data from the ArrayTube and the ArrayStrip platform in laboratory A and measured microarray raw data on the ArrayStrip platform in laboratories B and C.

## Data Availability

The datasets supporting the conclusions of this article are included within the article and its additional files.

## Abbreviations

AUC: Area under curve
CI: Confidence interval
ELISA: Enzyme-linked immunosorbent assay
FCI: Food chain information
OIE: World Organisation for Animal Health
ROC: Receiver Operating Characteristic
